# Hepatitis E virus is highly prevalent among pregnant women in Gabon, central Africa, with different patterns between rural and urban areas

**DOI:** 10.1186/1743-422X-5-158

**Published:** 2008-12-22

**Authors:** Mélanie Caron, Mirdad Kazanji

**Affiliations:** 1 Departement de Virologie, Centre International de Recherches Médicales (CIRMF), BP 769, Franceville, Gabon; 2 Service de Coopération et d'Action Culturelle, French Embassy, BP 2105, Libreville, Gabon; 3 Réseau International des Instituts Pasteur, Institut Pasteur, 28 rue du Dr Roux, 75015 Paris, France

## Abstract

Hepatitis E virus (HEV) is highly endemic in several African countries with high mortality rate among pregnant women. Nothing is known about the circulation of this virus in central Africa. We evaluated therefore the prevalence of anti-HEV IgG in samples collected from pregnant women living in the five main cities of Gabon, central Africa. We found that 14.1% (119/840) of pregnant women had anti-HEV IgG. The prevalence differed between regions and between age groups. In 391 newly collected samples from the region where the highest prevalence was found, a significant difference (*p *< 0.05) in seroprevalence was found between rural (6.4%) and urban (13.5%) areas. These data provide evidence of a high prevalence of HEV in Gabon, providing indirect evidence of past contact with this virus.

## Findings

Hepatitis E virus (HEV) is an enterically transmitted pathogen and is responsible for recent large-scale epidemics of hepatitis around the world, as reported recently in Uganda , where more than 7500 cases were registered in 1 year [1]. HEV induces self-limiting or acute hepatitis, and the severity can varied from no symptoms to fulminating infection [2]. HEV infections have not been known to become chronic [2]; however, recently, persistent HEV infection, with chronic hepatitis and cirrhosis, has been reported in patients with reduced immune surveillance induced by chemotherapy or post-transplant immune suppression [3,4]. The average mortality rate from HEV infection is 1–4%, principally among adolescents and young adults, but it is still not clear that the severity is age-dependent. For unknown reasons, the mortality rate is higher among pregnant women, especially during the third trimester [5]. In Sudan, a case:fatality ratio of 17.8% was found in an outbreak in Darfur, with a ratio of 31.1% among pregnant women [6].

In endemic areas, which include Africa, Asia and the Middle East, HEV outbreaks are waterborne, whereas in non-endemic areas such as Europe, Japan and the USA, sporadic cases of acute hepatitis are usually due to zoonotic foodborne transmission [7]. Bloodborne and perinatal transmission could also occur, but ingestion of fecally-contaminated water remains the main route of HEV transmission. Many HEV outbreaks have been observed in Africa, such as in Ethiopia and Somalia in 1988–1989, Djibouti in 1993, Morocco in 1994, Chad and Sudan in 2004–2005, the Democratic Republic of the Congo in 2006 and Uganda in 2007–2008 [1,8-12]. In the absence of outbreaks, the HEV prevalence in rural populations was 4.4% in Ghana, 14.0% in Burundi, 15.3% in South Africa and 67.7% in Egypt, with a seroprevalence of up to 84.3% among pregnant women [13-16]. There appear to be considerable differences in exposure to HEV in endemic areas.

Few data are available on the circulation of HEV in central Africa. In 1995, no anti-HEV IgG was found in samples collected in Libreville, the capital of Gabon [17], but the study was based on a small sample and did not reflect the actual situation in the country. Furthermore, the laboratory techniques for HEV detection have advanced considerably since the time of that study. The aim of the study reported here was to evaluate the prevalence of anti-HEV IgG in samples collected from pregnant women living in the five main cities of Gabon. We also compared the HEV prevalence in rural and urban areas in the region with the highest seroprevalence.

Gabon is located on the Gulf of Guinea near the Equator, and tropical forest covers three quarters of the territory. To evaluate the HEV prevalence among pregnant women, two epidemiological surveys were conducted. The first was conducted from January to March 2005, when blood samples were collected from all 840 pregnant women (mean age, 24.6 ± 6.4 years; range, 14–44 years) who attended a first antenatal examination in the five main cities of the country: Libreville, the capital city in the north-west; Port-Gentil, the main harbor and economic capital in the west; Lambaréné, in the centre of the country; Oyem in the north-east and Franceville in the south-east. The second study was conducted from January to June 2007 in rural and urban areas of Franceville, where the highest seroprevalence was found. The study obtained ethical clearance from the ethics committee; data on age and geographic origin were retained only after informed consent had been obtained.

To determine the anti-HEV IgG prevalence, we used the HEV (TMB) ELISA Kit (Genelabs Diagnostics, Singapore) according to the manufacturer's instructions. Serological status in relation to the age group and geographical origin of the pregnant women was analysed statistically by the chi-squared test with Yates correction, and prevalences and odds ratios were calculated. The corresponding 95% confidence intervals (CIs) were reported as measures of statistical significance. Analyses were performed with Epi-Info (version 6.04dfr, ENSP-Epiconcept-InUS, 2001).

Anti-HEV IgG were found in 119 of the 840 samples (14.1%). The seroprevalence varied substantially, from 10.2% to 20.8%, by region (Table 1). The highest prevalence was found in Franceville, where the risk for contracting HEV infection was twice as high as in the other cities (Table 1). The seroprevalence was highest in the younger (14–20 years; 14.8%) and older (31–44 years; 16.8%) age groups than in the others (Table 1). However, there was no statistically significant difference in prevalence by age group.

**Table 1:** Prevalence of anti-HEV IgG among pregnant women in the main cities of Gabon, central Africa, and in different age groups

Variable	Prevalence of anti-HEV IgG				
	
	No. positive/No. tested	%	95% CI	OR	95% CI
Main Cities					
Libreville	27/183	14.7	9.6–19.9	1.24	0.78–1.97
Port-Gentil	16/157	10.2	5.5–14.9	0.75	0.43–1.31
Lambaréné	39/304	12.8	9.1–16.5	0.99	0.66–1.49
Oyem	8/57	14.0	5.0–23.0	1.15	0.53–2.49
Franceville	29/139	20.8	14.1–27.5	2.05	1.29–3.26
					
Age range (years)					
14–20	40/269	14.8	10.6–19.0	1.26	0.84–1.90
21–25	31/230	13.5	9.1–17.9	1.08	0.70–1.68
26–30	20/174	11.5	6.8–16.2	0.87	0.52–1.45
31–44	28/167	16.8	11.2–22.4	1.49	0.94–2.36
					
All	119/840	14.1	11.8–16.4		

CI, confidence interval; OR, odds ratio

Despite the high HEV prevalence among pregnant women, principally in Franceville (Haut-Ogooué region), symptoms compatible with acute viral hepatitis were not found frequently. In order to confirm the previous observation, a second epidemiological study, was conducted among 391 pregnant women aged 25.9 ± 6.9 years (range, 14–45 years) in both rural and urban areas of the Haut-Ogooué region. Of all these newly collected samples, 39 (10.0%) were positive for anti-HEV IgG (Table 2). A significant difference (*p *< 0.05) in prevalence was found between urban (Franceville city; 13.5%) and rural areas (villages of the Haut-Ogooué region; 6.4%), with a 2.5 times higher risk for infection in urban than in rural areas (Table 2).

**Table 2:** Prevalence of anti-HEV IgG in rural and urban (Franceville city) areas in the Haut-Ogooué region of Gabon, central Africa

Area	Prevalence of anti-HEV IgG				
	
	No. positive/No. tested	%	95% CI	OR	95% CI
Rural	11/173	6.4	2.6–10.1	0.46	0.22–0.95
Urban	28/207	13.5	8.8–18.1	2.46	1.19–5.09
					
All	39/391	10.0	7.0–13.0		

CI, confidence interval; OR, odds ratio

We have shown that HEV is highly prevalent in Gabon, central Africa, although a study conducted in 1995 [17] concluded that the virus was not present in this country. The prevalence of anti-HEV IgG in this study was similar to those in endemic and epidemic countries in South-East Asia [18] and to that in south-west France, where autochthonous HEV infection is frequently recorded [19].

In the first epidemiological study, in Franceville, 29 of 139 pregnant women had antibodies to HEV (20.8%). In the second study, 28 of 207 women in the same city had HEV antibodies (13.5%). The difference in prevalence between the two studies was not statistically significant. The finding that the prevalence in the first study was higher than that in the second might be due to the fact that in the first study the samples were collected from two maternal centers in which medical care is free and therefore women of low socio-economic status frequently attend. In the second study, in order to determine the true HEV prevalence in the area, samples were collected from six sentinel centers, including clinics, hospitals, and maternity centers. The difference may therefore be due to a difference in the population studied and the higher HEV prevalence in people of low socio-economic status.

Our finding of a higher risk for HEV infection in urban than in rural areas suggests the presence of local risk factors; however, the source of contamination remains unknown. The population density, the absence of sewer systems, the consumption of bush meat and the presence of excreta of peri-domestic animals near habitations might also be risk factors [20-22]. Furthermore, the excreta of HEV-infected persons might be contaminated, again implicating the precarious sanitary conditions in cities in the country.

Until now, no epidemic or sporadic cases of HEV infection have been reported, despite the high prevalence of antibodies to this virus. Furthermore, no liver markers compatible with acute viral hepatitis were found that would indicate past HEV infection among these pregnant Gabonese women. Probably, the initial HEV infection occurred early in life, and, as with early childhood exposure to hepatitis A virus in countries where it is highly endemic, the children do not become ill. Therefore, epidemiological studies of people in various age groups and in children are also needed.

Screening pregnant women for HEV is thus important for improving knowledge about the epidemiology (transmission and circulation) of this virus. More extensive studies should be conducted to evaluate the seroprevalence, to characterize the circulating HEV genotypes and to determine the current pathological and risk status in the general population of central Africa.

## Competing interests

The authors declare that they have no competing interests.

## Authors' contributions

MC carried out the serological studies and performed the statistical analysis. MK designed the study and drafted the manuscript. Both authors read and approved the final version of the manuscript.
